# Biological Activation of Inert Ceramics: Recent Advances Using Tailored Self-Assembled Monolayers on Implant Ceramic Surfaces

**DOI:** 10.3390/ma7064473

**Published:** 2014-06-12

**Authors:** Frederik Böke, Karolina Schickle, Horst Fischer

**Affiliations:** Department of Dental Materials and Biomaterial Research, RWTH Aachen University Hospital, Pauwelsstrasse 30, 52074 Aachen, Germany; E-Mails: fboeke@ukaachen.de (F.B.); kschickle@ukaachen.de (K.S.)

**Keywords:** alumina, bioactivation, self-assembled monolayers, tissue-integration, high-strength ceramics

## Abstract

High-strength ceramics as materials for medical implants have a long, research-intensive history. Yet, especially on applications where the ceramic components are in direct contact with the surrounding tissue, an unresolved issue is its inherent property of biological inertness. To combat this, several strategies have been investigated over the last couple of years. One promising approach investigates the technique of Self-Assembled Monolayers (SAM) and subsequent chemical functionalization to create a biologically active tissue-facing surface layer. Implementation of this would have a beneficial impact on several fields in modern implant medicine such as hip and knee arthroplasty, dental applications and related fields. This review aims to give a summarizing overview of the latest advances in this recently emerging field, along with thorough introductions of the underlying mechanism of SAMs and surface cell attachment mechanics on the cell side.

## 1. Introduction

Human life expectancy has increased tremendously over the last century, leading to a demographic shift especially in the western hemisphere. This has had major implications on the employment and long-term requirements of passive ceramic medical implants, such as total artificial hips (TAH), knees (TAK) as well as other substitution areas where ageing tissue like bone is prone to failure due to excessive wear and decreasing regeneration. Ceramics materials involved for such applications have ever since been a focus of constant research towards mechanically superior and longer lasting compositions to combat the challenges arising from the ageing recipient population. Ceramics as materials for full replacement of joints have been actively incorporated into this field since the early 1930s [1], although actual substrate compositions were not suitable for higher load bearing joint replacements, e.g., in the hip region, until 30 years later [2]. The most important ceramics for load bearing applications today are alumina (Al_2_O_3_) and tetragonal zirconia polycrystals (TZP), as well as dispersions thereof, primarily alumina-toughened-zirconia (ATZ) and zirconia-toughened-alumina (ZTA), all of which show excellent mechanical properties and a very high resistance to wear [3,4]. In the beginning, these high-strength ceramics were assumed to be somewhat biocompatible, based on their hydrophilicity, very low cytotoxicity and excellent corrosion resistance [5,6,7,8,9]. However, as numerous studies on all-ceramic prostheses in direct contact to e.g. bone tissue have shown, they are in fact almost completely bioinert, resulting in very limited interaction between the material and the surrounding tissue [10]. Over time, numerous attempts have been undertaken to activate a ceramic surface in order to increase its biological activity. Most of these follow the strategy to coat the surface of the implant with a bioactive layer, such as hydroxyapatite (HA), other calcium phosphates, bioactive glasses or composites thereof, which have all been shown to bond to bone [7,11,12,13,14,15,16,17,18,19,20,21,22,23,24]. These materials form a material-tissue interface with substantial resistance to impacting mechanical force, making the connection suitable for bonding purposes. However, the crucial weakness of these coatings is the interface towards the underlying ceramic material. Due to the different material properties, such as thermal expansion coefficients, hardness, brittleness and overall wear resistance, relative movements can occur at their intermediate border, resulting in a loss of adhesive strength of the coating after thermal treatment [25]. This movement eventually leads to a decrease in function until failure of the implanted device is inevitable. Debris particles, shed loose by the relative movement, along with the resulting fibrous coating responses from the surrounding tissue towards the generated friction, generally promote aseptic loosening [26,27,28,29,30,31].

The issue of aseptic loosening was one of the reasons to investigate ceramic-ceramic bearings in the first place, as other combinations, such as metal-metal, ceramic-UHMWPE (Ultra-High-Molecular-Weight-Polyethylene) and combinations thereof show significantly increased wear debris in comparison. Nowadays, the gold standard for dental, orthopaedic as well as related implant devices is a second engulfing cup of titanium, oftentimes an alloy of Ti6Al4V, on the chosen e.g., acetabular liner in the case of hip implants. A small overview of combination possibilities for this type of implant is shown in Table 1, displaying the limited choice for a tissue interface cup for bioactive bone-bonding [32].

However, the use of a titanium or stainless steel cup comes with the disadvantage of the aforementioned two components approach. Therefore, a direct functionalization of the ceramic, and thus reducing the implant to a single material while still promoting tissue-interaction, would be highly beneficial. In general, the lifetime of a hip joint replacement implant used e.g., in total hip arthroplasty ranges between five and 15 years, depending on the condition of the patient, the materials used, their respective combination and the exercised caution during implantation [33]. To minimise the need for revision surgery, and increase the patient’s quality of life, it is therefore necessary to explore alternatives in terms of bioactivation of ceramic implants to incorporate the implant as flawlessly as possible into the tissue, without risk of any of the disadvantages mentioned above.

A new technique for this activation of high-strength ceramics, without the introduction of an extensive coating, is the method of activating the surface through the attachment of Self-Assembled-Monolayers (SAM). This procedure has received immense focus over the last decades, although almost exclusively for the chemical surface modification of gold and other metal substrates [34]. The developing approach of using the layer-technique to bind bio-molecules or tissue-growth promoting cells and therefore activate the otherwise inert ceramic surface has not been investigated until recently [35,36]. This review therefore aims to give a condensed overview into the field of SAMs for the bio-activation of ceramics in healthcare with a clear focus on the most recent discoveries and the consequent possibilities and implied limitations thereof. Furthermore, a cautious outlook of the advantages accrued by this technology is provided.

**Table 1:** Current most popular and deployed combination alternatives for total hip replacement surgery [37]. Titanium alloys mostly used are Ti_6_Al_4_V. Ceramics are split between yttria-stabilized tetragonal zirconia polycrystal (TZP), Alumina (Al_2_O_3_), and their dispersion ceramics alumina-stabilized zirconia (ATZ) and zirconia-stabilized alumina (ZTA). For a more in depth discussion of these ceramics please refer to [32].

Component	Material class	Most used material
Femoral stem	metal	CoCrMo-wrought, Ti-alloys, stainless steel
Femoral head	metal	CoCrMo-cast, stainless steel
	ceramic	Alumina (pure or zirconia-toughened), zirconia
Acetabular cup liner	polymer	UHMWPE, XLPE
	metal	CoCrMo-cast
	ceramic	Alumina (pure or zirconia-toughened), zirconia
Acetabular cup shell	metal	Commercially pure titanium, stainless steel

## 2. Chemical Activation of Inert Ceramic Surfaces

As described above, high-strength medical-grade ceramics are bioinert, yet possess unique material properties which make them excellent implants for various medical applications. An activated surface, without the supplement of an additionally engulfing material, would therefore greatly increase the ceramics applicability and deployment areas. As shown previously, the desired bioactivation can be achieved by activation of the surface alone, without alteration of the underlying substrate. Therefore, several investigations into possible modification strategies were undertaken over the last couple of years. This chapter is aiming to give a general overview over the early stages of research so far. Furthermore, a more in-depth discussion of the most recent, although quite limited, results from the newly surfacing strategy using SAMs as a multi-purpose layer for subsequent biological activation is given, along with a brief introduction into the relevant portions of the SAM research thus far.

### 2.1. Direct Surface Modification on Ceramic Substrates

Investigations into silane to alumina binding using Rayman spectroscopy on a not silver (Ag) enhanced surface were first carried out by Thompson and Pemberton [38] in 1995. They examined the interaction between different octadecyl silanes as well as stearic acid applied directly to an alumina surface. Their findings suggest that a direct coupling of octadecylsilanes leads to a more disordered layer in comparison to a previously applied underlying silicate (Si/SiO_2_) or Ag layer. When extended by a dimethyl ((CH_3_)_2_) group, the disorder among the silane terminal ending was similarly high, most probably from the mutual volumetric interaction between the rather bulky methyl groups introduced on the surface. However, surface groups prepared with stearic acid were found to be comparatively ordered, which might result from the stronger binding affinity between the functional end group and the alumina surface, which suggests that the binding and layer quality is highly dependent on the prevalent conditions on the surface as well as the overall reactants used. Expanding on this idea, Lee *et al.* [39] examined the reaction and binding kinetics of methyl- and aminosilanes on alumina substrates, where each silane was again pre-synthesized into holding a specific terminal group. Furthermore, they attempted to create mixed monolayers from a competitive application of both silanes in a mixed solution. Their most important finding was that methylsilanes were absorbed twice as fast as the equally present aminosilanes, resulting in a variation of silanes between the solution and the produced monolayer. However, the adsorption itself was random, meaning that although the kinetics favour methylsilanes, its adsorption process is not preferred over that of aminosilanes.

In 2005, Fischer *et al.* [13] reported the successful direct binding of hydroxyl (–OH) functional groups onto alumina substrates without an attached silane carbon chain. After 24 h immersion in a bath of 1 M NaOH at 100 °C, the aluminium oxide exposed at the surface was modified to aluminium hydroxide [40], according to the following reaction:

Al_2_O_3_ + 2NaOH +3H_2_O → 2Na^+^ + 2[Al(OH)_4_]^−^ (1)

Although surface modification always bears the risk of greatly deteriorating the materials’ mechanical properties, subsequent tests showed no severe impact towards its characteristic strength, its Weibull modulus or subcritical crack growth behaviour. Cell culture tests conducted *in vitro* with osteoblast-like cells displayed an increase in adhesion towards untreated specimen of 36% and 24% after 24 h and 7 days, respectively. Furthermore, alkaline phosphatase (AP) secretion was not influenced whereas the overall protein content increased among the cells adhered to treated surfaces. Although these findings show the general possibility to directly functionalize an otherwise inert ceramic surface, the treatment itself is rather extensive with regard to the overall preparation process. However, similar findings were reported by He *et al.* [41], although on Zirconia toughened alumina ceramic foam. To investigate the influence of this treatment time, specimens were prepared to resemble porous grafts for bone tissue and subsequently immersed at 80 °C in 5 M NaOH solution for various periods of time. *In vitro* experiments with osteoblasts have shown that 5–10 h of immersion time is favourable in terms of AP secretion, cell proliferation and general cellularity. Albeit, conclusions have to be drawn carefully, as the change in multiple parameters, such as the investigated porous structure and the slightly altered substrate material, makes a direct comparison to the findings of Fischer *et al.* difficult. Besides hydroxylation, experiments have been performed to explore the possibility of binding carboxylic groups to an alumina surface as well. The activation process is hereby similar to the one applied previously. Bertazzo *et al.* [14] used an analogous heating method where alumina powder was stirred in 60 °C preheated dicarboxylic acid for 8 h and subsequently pressed into disks. When immersed in simulated body fluid (SBF), a precipitation of Ca^2+^ was observed, which subsequently lead to the formation of a calcium phosphate layer on the carboxylated surface. Osteoblast adhesion tests showed an increase of viability if adhered to the newly created calcium phosphate surface in comparison to an untreated negative control. The carboxyl-modified surface seems to play an important role in the adherence of this layer. As on untreated alumina substrates, this effect was only observed after increasing the concentration five-fold towards normal blood plasma [42]. Unfortunately, the specimens were merely pressed into disks, and not properly sintered and subsequently grinded and polished, which represents standard procedure for implants. This renders a translation of these findings very complex, as a subsequent heat treatment after functionalization might have a serious decomposing effect on the surface binding. In a follow-up experiment, the same group investigated the influence of different length carboxylic acid chains on the electric surface charges of these powders [43]. It was found that the surface isoelectric point (IEP) in an environment closest to the human *in vivo* pH can be achieved with a treatment of butanedioic acid, and a resulting chain length of four carbon atoms between the functional and the terminal end of the carboxylic acid. As described previously, the modification of the surface charge might be a significant factor in protein adsorption and general bio modification [44]. Therefore, through alteration of the carbon chain length, different adsorption properties might be achieved, resulting in a possibly large variety of options.

In 2007, Kaltenborn *et al.* [45] reported the successful coupling of phosphate groups to properly sintered and subsequently grinded and polished alumina samples. A precipitation of calcium phosphate after SBF tests suggests a bioactive layer, which may be expanded to a coupling method for amino acid sequences of proteins. However, the treatment necessary to bind the phosphate groups was extremely aggressive, as the sample was boiled in monoaluminiumphosphate solution at pH 1. SEM studies have subsequently shown a micro-porous surface structure, indicating an erosion process during treatment. Although mechanical tests were not performed, a deterioration of mechanical key properties is likely. On the other hand, a porous surface might promote bone ingrowth and therefore add to the bonding process, a feature that requires additional attention when investigating highly aggressive surface treatments.

### 2.2. Silane-Based SAM Systems

The application of Self Assembled Monolayers (SAM) was first described by Zisman *et al.* [46] in 1946 and has been a highly investigated research topic with numerous applications and manifold branches established today. This is especially noteworthy, since the underlying strategy has hardly been altered over the decades, yet new applications are discovered and implemented still. This can mostly be attributed to the virtually limitless synthetic capabilities of organic chemistry and the accompanied diversification and modification possibilities of the attached terminal ends of the created SAM. It is therefore beneficial within the scope of this manuscript to give a condensed insight into the SAM technique believed to be most promising for the surface modification of high-strength ceramics.

**Figure 1:**
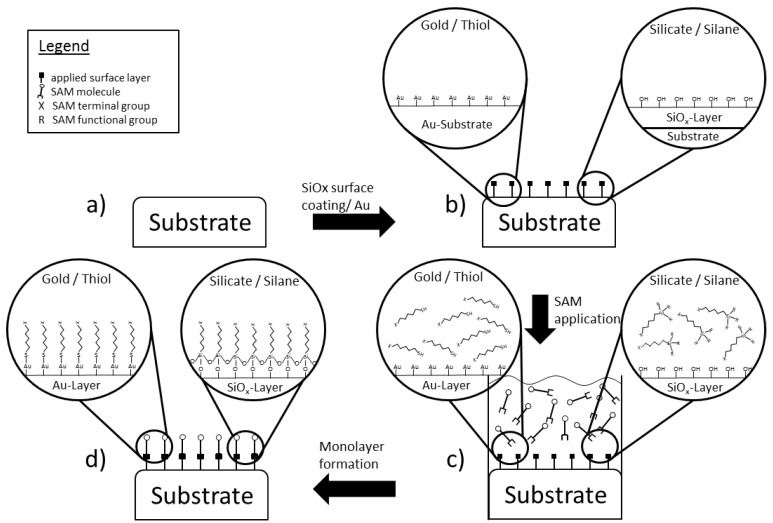
The general assembly scheme for the generation of a Self-Assembled Monolayers (SAM) on an inert surface is depicted with respect to a silicate-silane and a gold-thiol monolayer assembly. Please keep in mind that this scheme is meant to serve as a very abstract joint representation for two completely independent complex processes. (**a**) An originally inert surface is unable to house any further surface modification without prior activation; (**b**) by depositing a layer of Si/SiO*_x_* onto the ceramic surface, this additional activation becomes possible; (**c**) the SAM itself is applied through the addition of a solution holding the desired molecules in such a way that a surface attachment is only possible in one specific direction; (**d**) after the reaction, the solution is retracted and an originally inert surface has been chemically activated and is capable of housing further molecules of various kinds.

In general, SAMs consist of a variety of pre-prepared micro-structures, usually present in a form of liquid solution, which align themselves autonomously due to the prevalent chemical or physical conditions found at the attachment site (Figure 1). The result is a covalently attached layer, comparable to a form of carpet, which allows for further functionalization on its upward facing terminal end. This chemical reactiveness on the outward surface can be influenced by specific tailored head and tail ends of the structures in the applied solution. By therefore carefully composing the mixture of substrate, SAM-solution and subsequent terminal functionalization, a multitude of subsequent molecule adhesion is feasible. From the variety of different readily available SAMs today, the most popular ones are thiols (R–SH) on a gold substrate and silane-based (Si*_n_*H_2*n*+2_) systems on a Si/SiO_2_ substrate, both with varying alkyl chains lengths [34,47]. As mentioned above, the silane-based approach will be discussed with slightly more detail, as it has been shown to comprise desirable properties for ceramic functionalization [35,36].

SAMs based on silane-coupling (Si–O–Si) require a surface either already equipped with, or modified to contain, silicate (Si/SiO_2_) groups [48]. For other purposes, this feat is already accomplished by selection of a proper substrate, in most cases a material with silicon already incorporated. In order to obtain these structures on the otherwise inert high-strength medical-purpose ceramics however, alternate procedures have to be found. Schickle *et al.* [35] came to the conclusion that physical vapour depositioning (PVD) is a suitable, reproducible method to produce a reliable silicate layer onto a ceramic substrate as opposed to e.g., flame pyrolysis, which showed an incomplete and a qualitative irreproducible coating. Although the chemical bond between layer and substrate is of ionic nature, tensile tests have indicated that a sufficient strength can be applied without signs of detachment.

Once this silicate layer is applied, it serves as a foundation for further chemical procedures. When exposed to a humid environment, the –Si terminal groups become hydroxylated (–SiOH), which in turn serves as a reaction basis for the attachment of various alkylsilanes, e.g., alkylchlorosilanes, alkylalkoxysilanes, or alkylaminosilanes [34]. Here, the aforementioned hydroxyl group binds to the silane candidate through covalent Si–O–Si bindings. This process has been examined thoroughly over the last decades and has led to the attachment of silane monolayers on aluminium oxide [49,50], although without prior silicatization, as well as non-ceramics such as gold [51,52], silicon oxide [48,49,53,54,55], and quartz [56], among others. Due to the requirement of a hydroxylated surface, the amount of water present in the surrounding environment, solution or atmosphere during functionalization is critical for the overall quality of the monolayer [53,57,58]. Indeed, a complete covering layer can only be formed on sites where complete hydroxylation took place before [48,54]. In contrast, excess amounts of water, *i.e.*, more than necessary for a fully hydroxylated surface, may result in premature polymerization of the silane still in solution, and subsequent precipitation of the then formed polysiloxane chains on the substrate, without prior sufficient adherence [56]. Furthermore, temperature has been reported to have an effect on the SAM formation, too, although the environmental conditions on the deployment site for a medical implant, 37 °C body temperature, is considerably higher than the reported critical minimal reaction temperature of 18 °C for octadecyl and 10 °C for tetradecyl chains [53]. As mentioned before, a crucial requirement for silane-monolayer formation is the nature of the underlying silicate chains structure. This is particularly important, as equilibrium has to be found between the optimal silane-binding conditions for the monolayer formation by hydroxylation, yet the increased higher surface hydration has been reported to lead to a greater disorder and more overall coating defects [59].

**Figure 2:**
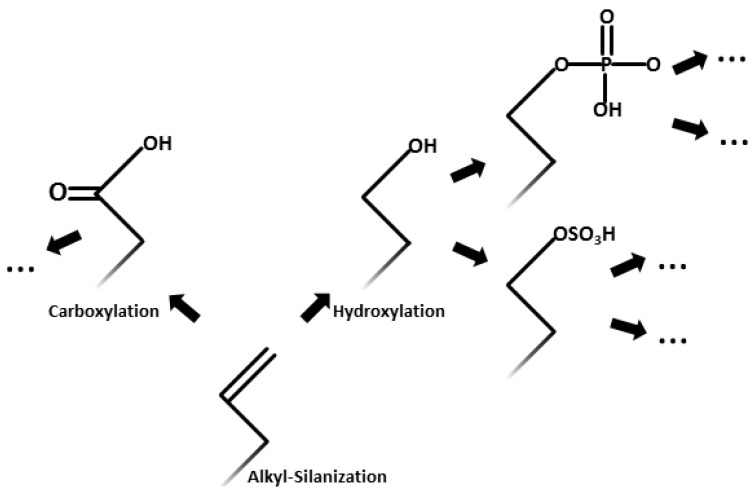
A selection of secondary functionalization following the alkylsilane alkene terminal groups on the surface is depicted. Note that these are only a very small selection of the applicable or even possible chemical reactions and only serve to point out the distinct potential inherent in this surface activation method.

Once established, the covalently bound silane monolayer can be further functionalized either by introduction of additional silane coupling agents already supplied with the desired terminal group or by application of additional chemical reactions. Using already terminally functionalized silane-coupling agents has shown to have the adverse effect of causing a high degree of disorder in the monolayer. As described above, this might result from the introduction of polar groups onto the otherwise non-polar alkyl chain and the resulting surface interactions [60]. Manifold silanes with a multitude of already attached functionalized terminal groups are available today, e.g., with halogen [61,62,63], methyl ether, acetate and thioacetate [61] or vinyl [48,53,64,65,66,67] groups, among others. Despite this already available diversity, for the aforementioned introduction of disorder, among other reasons, a more promising strategy seems to be the modification of their natural alkyl state through further chemical reactions instead of a preceding incorporation of the desired terminus on the silane. This approach exploits the almost limitless possibilities of modern organic chemistry, as the predominant alkene ending on most alkyl-silanes is very readily accessible for further modification. A limited selection of applicable functionalization is depicted in Figure 2. It is beyond the scope of this review to give a detailed insight into all possible monolayer applications. Therefore, for a more detailed discussion of the different properties and applications associated with the various self-assembled monolayers to date, refer to the detailed reviews of Ulman [34] and Schreiber [47].

### 2.3. Direct and SAM-Based Surface Modifications on Zirconia and Related Dispersion Ceramics

Although most of the surface functionalization methods described above are readily transferable to additional ceramics besides alumina, research conducted on other major ceramic implant materials, such as zirconia (ZrO_2_), shall be briefly discussed in more detail. Because of its excellent mechanical properties, zirconia has been a widely used implant material for a long time. However, due to an unusually high number of zirconia incorporating hip implants fractured and thus, the deployment of zirconia related products has decreased for applications outside the dental field, despite the evidence indicating a process related cause [68]. Nevertheless, surface modification research has been undertaken for a long time now to either purely enhance the material’s mechanical characteristics, or improve its tissue-integration capability.

In 1985, Legg *et al.* [69] implanted Al^+^ and Zr^+^ ions into the upper most layer of a zirconia disc-shaped sample by electron beam bombardment, in an attempt to enhance its mechanical properties. The treatment showed a successful implantation of foreign ions into the surface and a related increase in micro-hardness of up to 20% for the Al^+^ treated sample. However, this led to an increase in brittleness on the surface layer, which might render a possible implant more prone to internal failure. A different approach for incorporation of magnesia into the surface of yttria-stabilized tetragonal zirconia polycrystals (Y-TZP) was developed by Chatterjee *et al.* [70] who doped the material during sintering and through compressing stress while grinding and polishing the surface. Here, the material exhibited a hardened outer surface while simultaneously maintaining the phase inherited toughness. Unfortunately, no further studies were conducted to assess the modified layers cytocompatibility. To assure this basic biocompatibility of zirconia and zirconia-alumina composites, several studies have been undertaken. 

An investigation regarding the biocompatibility of alumina-zirconia composites was undertaken by Roualdes *et al.* [10], who detected no harmful influences on either proliferation, extra-cellular matrix secretion or cell morphology *in vitro*. However, during a subsequent *in vivo* study, it was found that the investigated osteo- and fibroblasts responded only very limitedly to the materials in terms of the aforementioned properties, suggesting a more thorough investigation towards the predominant conditions influencing the material after implantation. Further studies confirmed the general biocompatibility of zirconia towards osteoblasts [71], and even a beneficial reduction of bacterial adsorption in a dental milieu [72]. However, despite the sufficient biocompatibility, osseointegration of a mere surface treated zirconia sample, without further chemical modification, was found to be inferior to alumina and already established materials [71].

Therefore, in order to create the necessary surface for silane-coupling, Piascik *et al.* [73] treated the zirconia surface with a fluorinated plasma, to generate a 1–3 nm ZrO*_x_*F*_y_*-layer. This layer was designed to improve the chemical bonding towards a subsequent silane coupling for a stronger adherence to resins, but could be easily modified to conjugate cells as well. In contrast, a silica-silane coupling was developed by Lung *et al.* [74], where the zirconia surface was silicated through sandblasting and subsequently silanised. A variety of silanes was then hydroxylated and their influence on the surface properties measured. Although a difference in surface-roughness was measureable, depending on the applied silane, further biological adherence studies were unfortunately not performed. In an attempt to develop a more practical chemical modification of zirconia-based surfaces to ensure a strong bonding to silanes, Piascik *et al.* [75] deployed a silica-layer via vapour deposition of SiCl_4_ followed by a gas-phase H_2_O treatment for 15 min. The treatment resulted in a Si*_x_*O*_y_*-layer of an average thickness of 3 nm. The treated samples showed a comparable affinity towards subsequent bonded computer aided design/computer aided manufacturing (CAD/CAM) materials, which indicates that the previous deposition method might indeed be a potential technique to improve silane to zirconia coupling.

To ensure subsequent protein absorption onto any kind of zirconia-silane layer it has to be ensured that the adhered linking agent, a protein or peptide of some sort, is facing in the direction of the adjacent cell. However, when co-adsorbed onto a silicate surface, two different linking alkyl-chains were found to be prone to an increase of disorder on a zirconia surface than on a comparable silicon oxide [76]. This feature might indicate that in order to create an environment for more than one protein or cell type, the silane coating has to be planned carefully with respect to the cross-reaction between adjacent chains.

As shown, the feat of coupling silane to a silicate zirconia surface is indeed possible and has been done so for quite some time in the dental industry. However, a subsequent immobilization of protein or a straight-up coupling of cells has yet to be investigated further. As mentioned before, the main research regarding this overall topic so far has been conducted on alumina samples. Fortunately, the results found during alumina studies are likely to be carried over into other materials, such as zirconia and related alumina-zirconia composites.

### 2.4. Promoting Tissue Integration through Functionalized SAMs

The relative simplicity of further surface modification of the once assembled monolayer, or even prior, has recently opened new fields of research towards biomolecule and subsequent cell adhesion and differentiation. To investigate the influence of these specific surface characteristics, Faucheux *et al.* [77] applied alkylsilanes attached to different terminal functional groups, CH_3_, COOH, OH, NH_2_, Br, and vinyl, to glass and silicon wafers and determined the influence of wettability, layer thickness and roughness on the surface and later on protein adsorption. Reportedly, the applied silanes were capable of creating a molecular smooth surface with R_a_ values between 0.5 and 0.7 nm, with the exception of an isolated NH_2_ aminosilane. Furthermore, the protein adsorption on CH_3_, NH_2_, and COOH was superior to the recorded values on OH modified surfaces, which might result from its very hydrophilic nature and a possible corresponding adsorption inhibition [78]. When confronted with a competitive solution of two key adhesion proteins, vitronectin and fibronectin, only vitronection could be detected to have attached to the SAM. However, the lack of fibronectin might possibly be associated with preparation related problems [79]. Extended investigations towards the wettability of a different kind of SAM, using alkanethioles as opposed to silanes, suggests that cell adhesion is mainly driven by the surfaces’ wettability and only influenced in part by the attached functional group, the layers’ density as well as the type of cell desired to adhere [44]. Mixed monolayers of CH_3_/OH were found to have the highest adherence effect on human umbilical vein endothelial cells (HUVECs) with water contact angles of 40°, whereas CH_3_/COOH and CH_3_/NH_2_ showed highest affinity with angles of 60°–70°. The same mixed layers combined with epithelial HeLa cells show a maximum adherence at 50°. Finding the optimal surface conditions for even a single purpose surface therefore is not trivial and requires extensive treatment and specific testing.

Due to the innovative nature of SAM-facilitated tissue-integration of high-strength ceramic substrates, very little research data has been published yet. Only recently, Schickle *et al.* [35] investigated into the possibility of functionalizing alumina surfaces by a tailored SAM technique. To create a suitable underlying silicate layer, they proposed two distinct procedures, physical vapour depositioning (PVD) of a silicate layer under high vacuum conditions, and flame pyrolysis on the untreated substrate. The latter however proved inferior during mechanical tests (tensile adhesive strength 19.7 *vs.* 15.1 MPa), as well as showing an increase of surface impurities of up to 9%, most likely due to the manual nature of the application technique and very limited controllability and therefore reproducibility of the influence parameters. The actual functionalization was performed via subsequent application of amino-functionalized monolayers as well as akylsilanes and successive chemical alteration to achieve hydroxylated and carboxylated terminal groups. The integrity of this functionalization was proven successful by contact angle measurements to determine the wettability of the surface. In a follow-up investigation, bovine serum albumin (BSA) was immobilized as a model protein onto an extended chemically treated surface with the addition of –NH_2_ and CH_2_ groups [35]. Here, attachment of this protein required the extension of the existing layer by an EDC/NHS linker molecule. Further experiments revealed a gradient originating from the underlying surface in the order of =CH_2_ < –OH < –COOH = –NH_2_, where the amount of BSA bound to the surface structure was the same for COOH and NH_2_. Albeit a model protein was used, the potential possibility to use a Self-Assembled-Monolayer system on an initially functionalized ceramic surface was proven to exist. By binding various biomolecules to the functionalized surface, promotion of tissue-bonding and, more specifically to the originating issue, osseointegration of the substrate seems to be possible. The nature of the attached chemical group was shown to have an *in vitro* effect on the differentiation potential of neural stem cells as well [80]. Similar to the previous discussed findings, a trend among different functional groups towards migration of neural stem cells (NSCs) onto prepared glass surfaces was reported, again showing a strong potential towards amino functionalized surfaces. Furthermore, the differentiation path chosen by the attached stem cell seems to be influenced by the underlying substrate as well. Here, investigations showed that e.g., –SO_3_H promoted oligodendrocyte differentiation, whereas –NH_2_ favours neuronal differentiation. Although the underlying structure of glass and silicon wafers makes a seamless transition of these findings towards ceramic substrates difficult, it shows that an initial potential exists which might be further utilized in the upcoming studies.

## 3. Biological Interaction on the Functionalized SAM

As depicted above, recent advances and breakthroughs in the SAM-on-ceramics topic shifts attention towards the coupling of biomolecules and related proteins onto the terminal layer end for a promoted tissue-conduction as well as tissue-integration in general, and in terms of orthopaedics towards a specific osseointegration. Therefore a broad overview of the underlying mechanisms in osteoblast cell adhesion and differentiation in contact to biomaterials shall be given here. For a more detailed depiction, refer to the more specialized review of Anselme [81].

### 3.1. Osteoblast Adhesion Strategies for a Bioactivated Surface

Briefly, the adhesion process can be segmented into two distinct phases. In the first phase, osteoblasts attach to the surface and spread out until a sufficiently complete adhesion is achieved. The successive phase is governed from a motivation to proliferate and migrate across the surface until a continuous layer along the provided potential attachment points is reached. To achieve the adhesion during the initial phase, the osteoblast secretes a high number of collagenic proteins from which numerous are involved in the adherence process; additionally fibronectin has been shown to positively influence osteoblast adhesion, if present during the attachment process. If displayed competitively, osteoblasts seem to prefer adhesion towards fibronectin, as opposed to the collagen proteins [82]. As a way to recruit osteoblasts from their niche towards the surface in question in the first place, a number of chemotactic proteins have been investigated over the last decades. A promising candidate so far has been RGD (protein amino sequence: Arg-Gly-Asp), as it is able to fixate integrin, which has proven beneficial for the osteoblast attachment process [83]. Attempts to synthetically replicate the RGD structure to further improve relevant internal attachment sites have yielded several similar structures, yet studies have not shown superiority in their cell attachment properties so far [82,83,84]. This strongly suggests an attachment dependency on the molecules special confirmation, *i.e.*, its internal and special orientation and organisation.

From the bulk of adhesion molecules known to date, only cadherin and integrin are present in osteoblasts. However, since cadherin is mainly promoting cell–cell interaction, integrins are of primary interest for cell-to-molecule interaction. Integrins itself are build-up by different subunits, α*_x_* and β*_x_*, where *x* denotes the specific subunit. α 1–16 and β 1–8 can be combined to 22 heterodimers, all serving distinct purposes in the cells environment. Although certain similarities exist between cell types of a certain body region, only α_1_ and α_5_ are shared by all bone cell types [85]. Besides cell attachment, integrin has been shown to promote cell migration during the previously mentioned phase two as well [86], making it a versatile coupling molecule with enormous potential for the bioactivation of inert biomaterials. To access this potential though, the molecule has to be matched in an orientation favourable for the inbound cell, *i.e.*, facing the cell with its substrate binding cavity outwards [87]. This orientation is dependent on the underlying surface topography, chemistry and energy, of which the latter two can be properly adjusted by careful chemical manipulation of the terminal endings of the SAM [34]. Even minor deviations from the optimal constellation in surface chemistry have been shown to produce quite different results, as the cell itself is capable of discriminating between vanishingly low differences in surface attributes [88,89,90,91,92]. This has been shown for differencing surface charges as well, as protein adsorption onto various polymers seems to differ heavily when changing from a positive charge to overall negative surface energies [93]. As previously mentioned, the energy and chemistry present on a SAM can be adjusted towards favourable values, yet surface topography below has to be fine-tuned prior to any functionalization. This is quite important, as the grade of cell attachment is correlating with the degree of roughness present on the surface [94]. Cell spreading and subsequent continuous cell-layer formation has been described to be superior on smooth surfaces in comparison to rough ones [90,95,96]. However, the amount of possible surface topography modification is limited as it is critical to preserve the ceramic substrate mechanical properties [32]. An alternative to osteoblast adhesion could be the chemotactic recruitment of osteoblast-progenitor cells, e.g., human mesenchymal stem cells, hMSCs, onto the surface and their subsequent differentiation thereupon. Experiments performed on human bone-derived cells cultured on titanium based substrates have implied a strong differentiation potential of the cells depending on the underlying structure topography [97,98]. Therefore, proteins currently under investigation for the improvement of differentiation of osteoblast-like cells on modified surface chemistries are chemotactic growth factors such as members of the TGF family, e.g., β_1_, as well as BMP adhesion proteins. The latter ones have additionally been proven to positively influence the process of osseoconduction itself [99,100,101,102,103].

### 3.2. Influence of the Underlying Surface Chemistry and Immobilized Proteins on Endothelial Cells

As depicted above, the possibilities for further modification of the terminal group of the attached silane are enormous. Therefore, an attachment to many other cell types besides osteoblasts might be feasible by a careful pairing of an immobilized ligand on a surface with a specific receptor of the desired cell. As endothelial cells are necessary components of almost any kind of tissue integration, due to their involvement in the tissues’ vascular supply, their potential immobilization on an implant surface utilizing a SAM-transferable technique shall be discussed in more detail.

In order to promote a desired response in terms of adhesion, differentiation and proliferation on the implant surface, certain requirements have to be met and, even more importantly, carefully crafted around each other. Hereby, a first crucial step is the selection of the right silane to later couple the cell to. To better understand the diverse reaction patterns expressed by human umbilical vein endothelial cells (HUVECs), Kapur and Rudolph seeded them on two different kinds of silanes, EDA and PEDA [104]. It was found that the underlying surface chemistry of these modifications had a significant impact on the cellular morphology. When subjected to an additional external hydrodynamic flow, the cells seeded on EDA showed a higher resistance against detachment, suggesting a tighter bond to the underlying silane. Furthermore, the cell spreading of cells immobilized on EDA was increased in comparison to the PEDA surface, which might result from the higher free surface energy of the former, and the hydrophobic nature and incorporated phenyl group in the latter. They conclude, that besides the favourable coupling of endothelial cells on EDA, a use of well-defined monolayer systems comprising of different organosilanes might even aid in the creation of a more complex environment such as an extracellular matrix, but only if the separate building blocks are chosen carefully.

When the underlying chemistry is defined and properly selected, the next step is to incorporate cell attachment proteins to further enhance the affinity between cell and surface. Here, a protein-specific adsorption to the surface might promote not only adhesion but proliferation and cell production as well. In 2002, Balcells and Edelmann investigated the potential of pre-adsorbed fibronectin, laminin and gelatin to stimulate these impacts on bovine aortic endothelial cells (BAECs) [105]. The results of their experiments showed, as expected, that these mediators do indeed stimulate BAEC responses differently, depending on the kind of immobilized protein, as long as the cell-site binding specific α_5_ integrin was not blocked. Although their experiments were performed on tissue culture polystyrene (TCPS), the results should be applicable to other surfaces, such as SAMs, with conjugated proteins as well.

Lastly, the kind of immobilized protein is of high importance to the adhesion of endothelial or related cells. Studies performed with the Arg-Gly-Asp (RGD) peptide showed that there are definite differences between the conformations even of the same protein. In an experimental setup to mimic blood flow along endothelial cells, Xia and Truskey [106] showed that a switch to the cyclic form of RGD (cRGD) is of great benefit to the maximum endurable stress the cell can sustain before detachment from the substrate. They further suggest that the ligand density on the surface is directly correlated to the adhesion strength, thus making it more favourable to incorporate a higher amount of protein into the surface layer. In a similar study, yet without any protein on the surface, Spargo *et al.* [107] came to the conclusion that spatial control of endothelial cells on a SAM is a method to ensure a greater differentiation potential towards neovascular cords. They additionally showed that an incorporation of the wrong combination of protein and cell might have adverse effects, as a seeding of endothelial cells on immobilized heparin sulphate showed inhibition of cell growth even compared to the untreated silane.

## 4. Conclusions

Over the last decades, a huge amount of research focused on the application and improvement of self-assembled monolayers. Yet, only in the past decade this field has opened up possibilities for the biological integration of bioinert high-strength ceramics for the medical field. This paper aimed to give a broad overview on the latest developments of tissue-integration promoting surface modification and functionalization of these ceramics in general and for bone-implant purposes in particular. Alongside, a brief introduction into closely related fields was provided. Newly explored surface pre-treatment options have been introduced in terms of vapour deposition (PVD) of silicate layers on ceramics, enabling the potential succession in terms of chemical modification on these surfaces. Furthermore, these modifications have been shown to enable surface-molecule-protein bindings in principle, which gives reason to expect a potential tissue and, more specifically, osseointegration to be proven with actual cells in the future. However, it has become clear that the amount of published research data regarding this topic so far is sparse at best, mainly due to the very recent introduction of this general technique in ceramics.
